# Central adiposity and sex are associated with the integrative architecture of physiological networks in healthy youth: a cross-sectional study in a cohort of Chinese college students

**DOI:** 10.3389/fphys.2026.1859147

**Published:** 2026-07-16

**Authors:** Jun Wang, Juxia Chen, Juan Wu, Xuechun Ding, Juan Wang, Dongdong Zhu, Zhixiang Peng

**Affiliations:** Department of Medicine, Anqing Medical College, Anqing, Anhui, China

**Keywords:** early life cardiovascular risk, network physiology, obesity heterogeneity, sex dimorphism, waist circumference

## Abstract

**Background:**

The rising burden of cardiometabolic disease necessitates understanding how obesity and sex are associated with the integrative architecture of multi-system physiological networks in youth.

**Materials and methods:**

Using cross-sectional data from 3, 295 healthy Chinese young adults, we characterized the integrative physiological network architecture using Gaussian graphical modeling (EBICglasso). Network comparison tests, bootstrap resampling, and sensitivity analyses evaluated the effects of sex and different obesity indicators on network topology.

**Results:**

The analysis revealed a stable three-module network comprising body composition, autonomic, and cardiac subsystems. Waist circumference emerged as the most central node, exhibiting significantly higher expected influence (1.582) than body mass index or body fat percentage. Despite comparable global strength, distinct sex differences were observed: males displayed 3.123-fold stronger within-electrocardiographic module connectivity, whereas females demonstrated stronger cross-module connectivity between autonomic and cardiac systems. Obesity effects showed indicator heterogeneity: body mass index- and body fat percentage-defined obesity generally attenuated network connections, while waist circumference-defined obesity exhibited a distinct, non-attenuating pattern in females.

**Conclusion:**

This study delineates a multi-system physiological network architecture in healthy Chinese youth, establishing waist circumference as the most central node and revealing that obesity associations with network topology differ by both anthropometric indicator and sex. These findings highlight the potential of integrative system-level physiology to complement isolated risk metrics for early cardiometabolic risk stratification.

## 1 Introduction

The increasing global burden of cardiometabolic diseases, accelerated by the obesity epidemic, underscores the need to identify early physiological alterations that precede clinical onset (Andersson and Vasan, 2018; Busetto et al., 2024). Conventional physiological and epidemiological research has largely relied on a reductionist approach, examining individual or pairwise associations between biomarkers (Börgeson et al., 2024; Rubino et al., 2025). While informative, studies focusing on isolated metrics, such as anthropometrics [e.g., body mass index (BMI), waist circumference (WC)], autonomic nervous system activity [e.g., heart rate variability (HRV)], or cardiac electrophysiology (e.g., QT interval), inherently cannot capture the complex, integrated functioning of multiple physiological systems.

Non-invasive measures like BMI, WC, body fat percentage (BFP), HRV, and the corrected QT interval (QTc) are particularly valuable for risk screening in young, asymptomatic populations. Extensive evidence supports their bivariate relationships: central obesity with autonomic dysfunction (Windham et al., 2012), autonomic imbalance with ventricular repolarization abnormalities (Almeida et al., 2006; Omran et al., 2018), and systemic adiposity with cardiac electrical instability via distinct pathways (Gómez-Ambrosi et al., 2024). However, by deconstructing physiology into isolated associations, this traditional approach fails to elucidate how these systems interact as an integrated network. Are the observed links between obesity indices and cardiac electrophysiology mediated by autonomic function, or do they represent independent pathways? Does the physiological system organize itself into distinct functional modules, and if so, which specific obesity measure holds the most central influence within this network? The traditional framework is less suited to address these questions of system-level organization and control.

Network Physiology provides a powerful framework to overcome this limitation by modeling the body as a complex network of dynamically interacting physiological subsystems (Ivanov and Bartsch, 2014; Pollock, 2025). This approach uses statistical dependencies among variables to infer the system’s architecture, identifying central regulatory nodes and delineating how physiological subsystems coordinate their activities (Bartsch et al., 2012; Ivanov and Bartsch, 2014; Porta and Faes, 2016; Pollock, 2025). This paradigm has revealed how network topology reorganizes across physiological states like sleep and aging, offering insights beyond single biomarkers (Bartsch et al., 2012; Porta and Faes, 2016). Furthermore, initial evidence points to sex differences in network structure (Barajas-Martínez et al., 2021) and indicator-specific roles of adiposity measures within such networks (Aguas-Ayesa et al., 2024; Palumbo et al., 2025). Young adulthood is a critical period for establishing lifelong cardiometabolic trajectories, during which physiological systems may exhibit greater plasticity (Andersson and Vasan, 2018; Barajas-Martínez et al., 2021). Therefore, applying Network Physiology to this population can capture foundational, system-level regulatory patterns. Detecting subtle topological alterations in health could yield novel biomarkers for early cardiovascular risk stratification.

Based on this background, this study had three primary aims: (1) To characterize the modular architecture and identify hub nodes within the multi-system physiological network linking body composition, autonomic function, and cardiac electrophysiology in a large cohort of healthy Chinese youth; (2) To determine whether this network structure exhibits significant sex dimorphism; and (3)To investigate the specific, and potentially heterogeneous, effects of different obesity indicators (BMI, WC, BFP) on the network’s topology. By addressing these aims, we seek to delineate the early system-physiological framework that may underlie future cardiometabolic risk.

## 2 Materials and methods

### 2.1 Study design and participants

This cross-sectional observational study was conducted from February 20 to June 20, 2025, on the campus of Anqing Medical College in China. A hybrid sampling strategy was employed: cluster sampling of students from mandatory health science courses (*n* = 2, 298) combined with campus-wide voluntary recruitment (*n* = 1, 157). A total of 3, 455 students were initially recruited. After excluding 160 participants due to missing data or failure to meet inclusion criteria, the final analysis included 3, 295 healthy university students (845 males, 2, 450 females; mean age 19.58 ± 1.02 years; see **Supplementary Figure 1** for the participant screening flowchart). Exclusion criteria included a history of cardiovascular or cerebrovascular diseases, pregnancy, psychiatric disorders, use of medications potentially affecting measurement accuracy, inability to cooperate with measurements, and unwillingness to provide written informed consent. The study was conducted in accordance with the Declaration of Helsinki and was approved by the Ethics Committee of Anqing Medical College (Approval No. 2025-02-004). All participants provided written informed consent, and data were de-identified to protect privacy.

### 2.2 Variable definition, measurement, and quality control

WC (cm) and hip circumference (HC, cm) were measured using a standard tape. Weight (Wt, kg), height (Ht, cm), and fat mass (FM, kg, via bioelectrical impedance analysis) were measured using a smart ultrasonic height-weight measuring instrument (Yun Kang Bao CP30B, Shenzhen, China). The following obesity-related indices were calculated (Krakauer and Krakauer, 2012): BMI(kg/m²) = Wt/Ht²; BFP (%) = (FM/Wt) × 100%. To assess body shape risk independent of BMI, the A Body Shape Index (ABSI) was calculated as WC/(BMI^2/3^×Ht^1/2^). For ease of statistical presentation, raw ABSI values were multiplied by 1000 and reported in units of ×10^-3^.

Electrocardiographic (ECG) indices were acquired using a standard 12-lead ECG device (CONTE 8000G, sampling rate 1000 Hz, Qinhuangdao, China). Five-minute resting ECG data were collected in a supine position (paper speed: 25 mm/s, calibration: 10 mm/mV) during predefined time windows: morning (8:00 to <12:00), afternoon (12:00 to <18:00), and evening (18:00 to <22:00). The following parameters were extracted using the device’s proprietary software (Version V3.4.7, Wuhan, China): Heart Rate (HR, bpm), Standard Deviation of NN intervals (SDNN, ms), Root Mean Square of Successive Differences (rMSSD, ms), Low Frequency power (LF, ms²), High Frequency power (HF, ms²), LF/HF ratio, R-R interval (RR, ms), QT interval (ms), and QT dispersion (QTd, ms). All cardiac and autonomic nodes (HR, SDNN, rMSSD, LF/HF, QTd, QTc) were derived from the same 5-minute ECG recording. The corrected QT interval (QTc, ms) was calculated using the Framingham formula: QTc = QT + 0.154 × (1000 - RR), which offers superior correction accuracy within normal heart rate ranges (Baumert et al., 2016).

Exercise data (total data for the 3 months preceding ECG assessment) were collected via the smartphone application “Budaolepao” (Version 4.1.3, Leping Sports, Wuhan, China). The app utilizes the phone’s built-in GPS and accelerometer to record users’ walking/running activities, with data automatically uploaded to the cloud and exported. Total valid exercise distance (TVED, km) was defined by the software’s default algorithm: a single session required a minimum distance of 1.50 km, a pace between 5’00” and 12’00” per kilometer, and passing at least 2 checkpoints. Sessions ≤3 km had to be completed within 1 hour, and sessions >3 km but <10 km within 2 hours (including pause time); sessions exceeding these time limits were not counted as valid. TVED, measurement time period, and age were included as covariates in the analyses.

All measurements were performed by trained and certified personnel. Measurements took place indoors in a quiet environment maintained at 22-24 °C. Participants rested in a seated position for at least 10 minutes prior to measurement and had refrained from strenuous exercise, food intake, or stimulant consumption for at least 4 hours. The ECG device underwent routine initialization and self-check. Skin was cleaned with alcohol swabs, and electrodes were placed at standard 12-lead ECG positions. Baseline calibration ensured an amplitude > 0.5 mV, noise level < -60 dB, ≥ 95% analyzable normal-to-normal intervals, and ΔHR < 10 bpm. If participants questioned their height or weight results, measurements were repeated and the average was recorded. The device was calibrated according to the manufacturer’s instructions after every 50 participants. Data quality was reviewed bi-weekly; 10% of the raw data were randomly selected, and two internal medicine physicians independently calculated derived measures (e.g., RR interval, QTc). For ECG data, manual calculation involved selecting 3 consecutive sinus rhythm beats from each recording (preferably lead II, based on waveform clarity), excluding beats with premature contractions or atrial fibrillation. A difference ≤ 20 ms between the two physicians’ calculations was considered acceptable (Neumann et al., 2023); otherwise, data were re-measured, recorded, and re-checked before inclusion. To quantitatively assess the reliability of this manual process, the inter-rater intraclass correlation coefficient (ICC) was calculated, with a pre-specified standard of ICC > 0.90 indicating excellent reliability (Koo and Li, 2016).

### 2.3 Statistical analysis

This study employed Network Physiology methods to construct a multi-system physiological association network in healthy Chinese youth. Data quality control was first performed: the 10 core physiological indicators were winsorized at the 2.5th and 97.5th percentiles to handle outliers (Wilcox, 2017), and all continuous variables were standardized using Z-scores. Between-group comparisons of baseline characteristics used the Mann-Whitney U test (suitable for non-normally distributed data), complemented by calculation of the non-parametric effect size Cliff’s Delta (paired with Mann-Whitney U) and the parametric effect size Cohen’s d to comprehensively assess the magnitude of sex differences (Ellis, 2010). The suitability of the data for network analysis was assessed using Bartlett’s test of sphericity (Bartlett, 1950).

A sparse partial correlation network was estimated using the gaussian graphical model (GGM) with the extended bayesian information criterion graphical least absolute shrinkage and selection operator (EBICglasso) algorithm (Epskamp and Fried, 2018). The EBICglasso hyperparameter γ was set to 0.5 to balance network sparsity and sensitivity (Foygel and Drton, 2010), while controlling for covariates (age, measurement time period, TVED). Network topology was characterized by global strength, global efficiency, characteristic path length, and network density (Rubinov and Sporns, 2010). Community structure (modules) was detected using the Louvain algorithm (Blondel et al., 2008). Node importance was assessed via expected influence (EI) centrality (Robinaugh et al., 2016). Network stability was evaluated using a non-parametric bootstrap approach (1000 iterations) to calculate the correlation stability coefficient (CSC) (Epskamp et al., 2018).

Sex dimorphism analysis employed a weighted bootstrap resampling method (100 iterations) to balance sample sizes, followed by a permutation-based network comparison test (NCT, 1000 permutations) to compare global network structure, edge weights, and node centrality between sexes (van Borkulo et al., 2023). Obesity effects were analyzed based on Chinese-recommended cut-offs (Department of Medical Administration, National Health Commission of the People's Republic of China, 2025) to define obesity status (Male: WC ≥90 cm, BMI ≥28 kg/m², BFP ≥25%; Female: WC ≥85 cm, BMI ≥28 kg/m², BFP ≥30%. ABSI was not categorized due to the absence of established, population-specific obesity thresholds in current Chinese clinical guidelines).Bootstrap methods (500 iterations) were used to calculate 95% confidence intervals for the differences in network topology metrics between obesity and normal-weight groups (Friedman et al., 2008). Interaction effects between obesity status and sex, age, and TVED were tested using linear models (Aiken et al., 1991) (the technical covariate “measurement time” was not included here as it was already controlled for during network estimation). Sensitivity analyses included comparisons of QTc formulas, covariate adjustment strategies, and variations in the network sparsity parameter. Variable set sensitivity was assessed by re-estimating the network using simplified sets retaining only a single obesity indicator (WC, BMI, or BFP) alongside autonomic and cardiac variables. The modularity null-model comparison was performed by generating 1, 000 degree-preserving random networks and comparing their modularity distribution against the observed value. Network similarity was quantified using Pearson correlation (James et al., 2013).All statistical tests used false discovery rate (FDR, *α* = 0.05) correction for multiple comparisons. Effect sizes and between-group differences are reported with 95% confidence intervals (CI). Analyses were performed using R (version 4.3.0) with the bootnet, qgraph, and igraph packages (R Core Team, 2023). Python (version 3.12) was used for graphing and visualization (Python Software Foundation, 2023).

## 3 Results

### 3.1 Baseline characteristics

This study included 3, 295 participants (845 males, 2, 450 females). Analysis of baseline data (**Table 1**) revealed that, except for HR(*p* = 0.096) and SDNN (*p* = 0.055), all other variables showed statistically significant sex differences after FDR correction (*q* < 0.05). Males had significantly higher values for age, WC, HC, Ht, Wt, TVED, BMI, ABSI, and the LF/HF ratio, whereas BFP, QTc (Framingham-corrected), and rMSSD were significantly lower in males compared to females. Effect size analysis indicated that body composition indicators generally exhibited large sex differences (WC Cliff’s Δ = 0.561, Cohen’s *d* = 1.12; Ht Δ = 0.865, *d* = 2.16; Wt Δ = 0.585, *d* = 1.15; BFP Δ = -0.487, d = -1.02). In contrast, autonomic and cardiac electrophysiological indicators mostly showed small to medium effect sizes (QTc Δ = -0.285, *d* = -0.43; rMSSD Δ = -0.075, *d* = -0.07). Both effect size measures demonstrated a consistent pattern (see **Supplementary Table 1**). Statistical power analysis confirmed that the study design had sufficient power to detect meaningful group differences. Manual quality control performed on a randomly selected 10% of ECG data showed excellent inter-rater reliability (ICC for QT interval measurement = 0.918).

**Table 1:** Baseline characteristics of the study population by sex.

Variable	Median [IQR]			p	q	Cliff’s Δ(95% CI)	Cohen’s d
	Full sample (N = 3, 295)	Male (n = 845)	Female (n = 2, 450)				
Age (years)	19.00 (19.00–20.00)	20.00 (19.00–20.00)	19.00 (19.00–20.00)	<0.001	<0.001	0.096 (0.054, 0.137)	0.189
WC (cm)	71.50 (65.80–79.90)	80.70 (72.80–90.00)	69.50 (64.50–75.90)	<0.001	<0.001	0.561 (0.529, 0.595)	1.125
HC (cm)	93.80 (89.50–100.10)	97.10 (91.40–103.50)	92.80 (88.70–98.50)	<0.001	<0.001	0.261 (0.216, 0.301)	0.465
Ht (cm)	163.50 (159.00–169.50)	174.00 (169.50–177.50)	161.50 (157.50–165.00)	<0.001	<0.001	0.865 (0.846, 0.884)	2.155
Wt (kg)	59.00 (52.20–69.05)	70.30 (61.30–80.70)	56.20 (50.60–63.60)	<0.001	<0.001	0.585 (0.553, 0.615)	1.151
TVED (km)	117.76 (109.25–141.68)	145.57 (105.01–180.87)	114.44 (109.35–137.97)	<0.001	<0.001	0.304 (0.255, 0.357)	0.299
BFP (%)	25.40 (21.40–29.70)	20.70 (15.30–25.90)	26.50 (23.00–30.50)	<0.001	<0.001	–0.487 (–0.528, –0.446)	-1.024
BMI (kg/m²)	21.91 (19.79–25.06)	23.28 (20.50–26.76)	21.58 (19.59–24.38)	<0.001	<0.001	0.219 (0.181, 0.260)	0.391
ABSI (×10^-3^)	71.65 (68.31–75.11)	75.12 (72.03–78.22)	70.65 (67.51–73.49)	<0.001	<0.001	0.504 (0.464, 0.538)	0.822
HR (bpm)	75.00 (68.00–83.00)	74.00 (66.00–83.00)	75.00 (69.00–82.00)	0.096	0.101	–0.038 (–0.086, 0.010)	-0.054
SDNN (ms)	49.13 (37.58–63.73)	50.26 (38.19–67.21)	48.78 (37.34–62.80)	0.055	0.062	0.044 (–0.004, 0.090)	0.086
rMSSD (ms)	40.44 (26.77–59.70)	37.79 (23.88–56.71)	41.22 (27.54–60.33)	0.0011	0.0013	–0.075 (–0.121, –0.032)	-0.065
LF/HF (ratio)	0.87 (0.50–1.55)	1.34 (0.78–2.34)	0.76 (0.45–1.29)	<0.001	<0.001	0.365 (0.325, 0.408)	0.509
QTd (ms)	32.00 (22.00–44.00)	33.00 (22.00–49.00)	31.00 (21.00–43.00)	<0.001	<0.001	0.089 (0.038, 0.130)	0.232
QTc (ms)	399.00 (387.00–410.00)	390.00 (380.00–405.00)	401.00 (390.00–412.00)	<0.001	<0.001	–0.285 (–0.327, –0.239)	-0.426

All continuous variables are presented as median (IQR) due to non-normal distributions. TVED represents the cumulative distance over the 3 months preceding ECG assessment, serving as an objective metric of habitual physical activity level and cardiorespiratory fitness, in contrast to single-day measurements or self-reported questionnaires. ABSI, A Body Shape Index; original values were multiplied by 1, 000 for numerical stability, and the unit is expressed as ×10^-3^ to reflect the scaled metric.QTc interval calculated using the Framingham formula.Statistical analysis: Between-sex differences were assessed using the Mann–Whitney U test. q-values represent FDR-adjusted p-values using Benjamini–Hochberg procedure (correcting for 16 variables). Effect sizes: Cliff’s Δ (non-parametric effect size corresponding to Mann–Whitney U test)—positive values indicate higher values in males, negative values indicate higher values in females. Magnitude: |Δ| ≥0.474 (large), 0.33≤|Δ|<0.474 (medium), 0.147≤|Δ|<0.33 (small). Cohen’s d (parametric effect size)—|d|≥0.8 (large), 0.5≤|d|<0.8 (medium), 0.2≤|d|<0.5 (small). *Post-hoc* power analysis confirmed adequate statistical power for detecting medium effect sizes across all analytical scenarios. WC, Waist Circumference; HC, Hip Circumference; Ht, Height; Wt, Weight; TVED, Total Valid Exercise Distance; BFP, Body Fat Percentage; BMI, Body Mass Index; ABSI, A Body Shape Index; HR, Heart Rate; SDNN, Standard Deviation of NN intervals; rMSSD, Root Mean Square of Successive Differences; LF, Low Frequency power; HF, High Frequency power; QTd, QT dispersion; QTc, Corrected QT interval; IQR, Interquartile Range; False Discovery Rate; CI, Confidence Intervals.

### 3.2 Full-sample network construction

**Figure 1:**
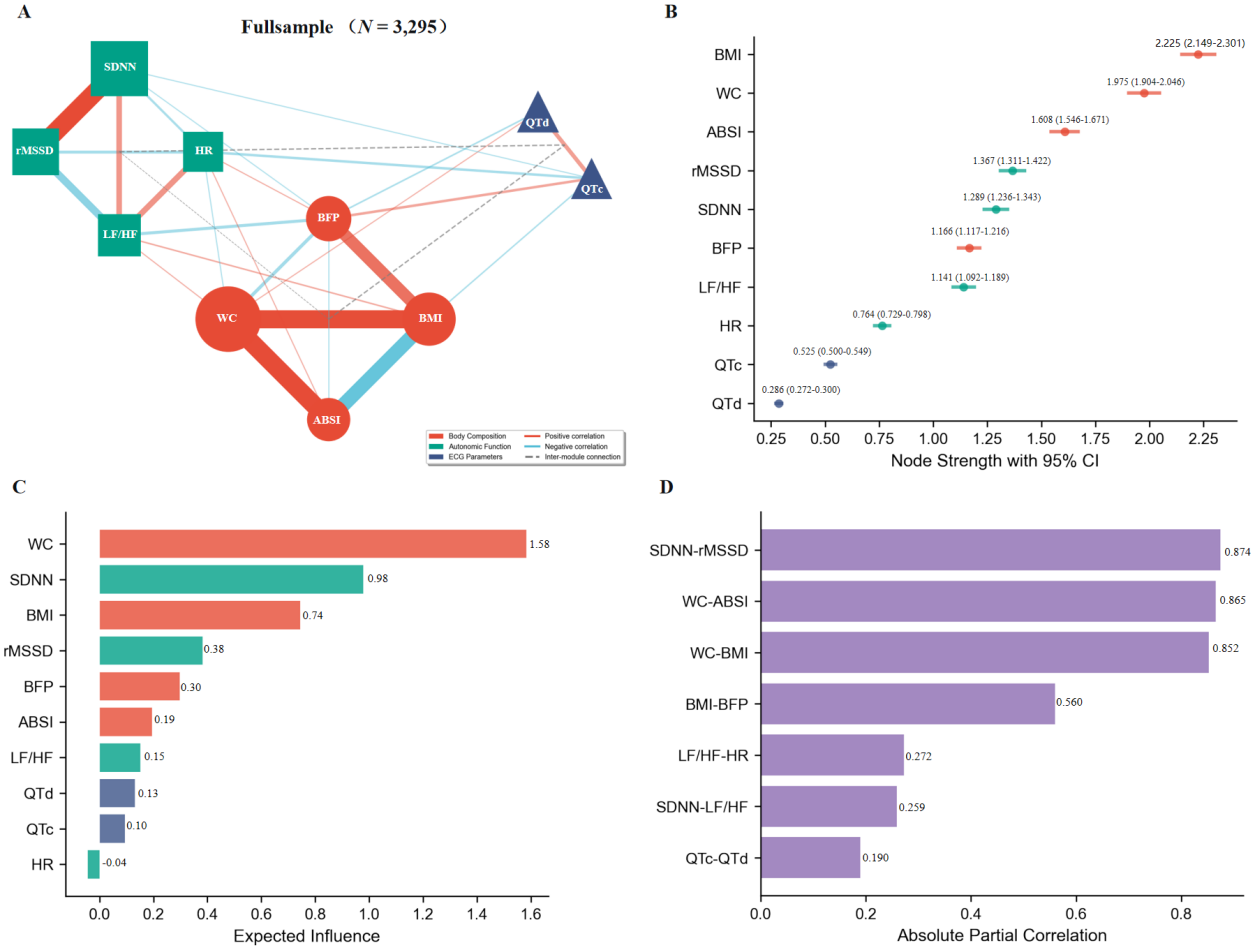
Multi-system physiological network architecture in Chinese healthy youth. **(A)** Full-sample network structure showing three distinct functional modules (Body composition, Autonomic function, ECG parameters) with inter-module connections. Only edges with absolute partial correlation > |0.01| are displayed. Edge width is proportional to connection strength, with red indicating positive partial correlations and blue indicating negative partial correlations. Dashed lines between modules represent inter-modular connectivity, with width scaled to connection strength. **(B)** Node strength stability with 95% CI from bootstrap analysis (CSC = 0.750), demonstrating high network reliability. **(C)** Node expected influence centrality ranking, showing WC’s superiority in centrality while BFP demonstrates higher connectivity. **(D)** Key intra-module connections showing the strongest partial correlations within each functional module. WC, Waist Circumference; BMI, Body Mass Index; BFP, Body Fat Percentage; ABSI, A Body Shape Index; ECG, Electrocardiographic; HR, Heart Rate; SDNN, Standard Deviation of NN intervals; rMSSD, Root Mean Square of Successive Differences; LF, Low Frequency power; HF, High Frequency power; QTd, QT dispersion; QTc, Corrected QT interval; CSC, Correlation Stability Coefficient; CI, Confidence Intervals.

Bartlett’s test of sphericity indicated significant overall correlations among variables (*p* < 0.001), supporting the suitability for network analysis. The sparse partial correlation network estimated using the Gaussian Graphical Model with the EBICglasso algorithm (tuning parameter *γ* = 0.5) comprised 10 nodes and 26 non-zero edges, with a network density of 0.58 and a global strength of 6.173. This network exhibited a clear three-module structure (**Figure 1A**), with a modularity Q value of 0.118. Module 1 contained all four body composition indicators (BMI, BFP, WC, ABSI) and showed the strongest within-module connections (partial correlations: WC–BMI = 0.852, WC–ABSI = 0.866; see **Supplementary Table 2**). Module 2 contained three HRV indices (SDNN, rMSSD, LF/HF) and HR, with the strongest connection being SDNN–rMSSD (0.874). Module 3 contained two cardiac electrophysiological indices (QTc, QTd), with a partial correlation of 0.189. Positive overall connectivity was observed between the ‘Body Composition–Autonomic Function’ modules (connection strength = 0.0023), while connectivity between the ‘Autonomic Function–ECG Parameters’ modules was stronger (0.0075). In contrast, direct connections between the ‘Body Composition–ECG Parameters’ modules were minimal. Network stability analysis indicated high reliability, with a non-parametric bootstrap CSC of 0.750 (95% CI: 0.716–0.784). A node strength stability plot (forest plot) further confirmed the reliability of individual node strengths (**Figure 1B**). Hub analysis (**Figure 1C**) revealed that WC had the highest Expected Influence (EI = 1.582), significantly higher than BMI (EI = 0.744; difference = 0.838) and BFP (EI = 0.297; difference = 1.285). The centrality ranking among obesity indicators was WC > BMI > BFP > ABSI. Analysis of within-module connections (**Figure 1D**) showed that WC–ABSI was the strongest connection within the body composition module (0.866), SDNN–rMSSD was the most prominent within the autonomic module (0.874), and BFP was the only obesity indicator showing a positive connection with QTc (0.120).

### 3.3 Sex dimorphism analysis

**Figure 2:**
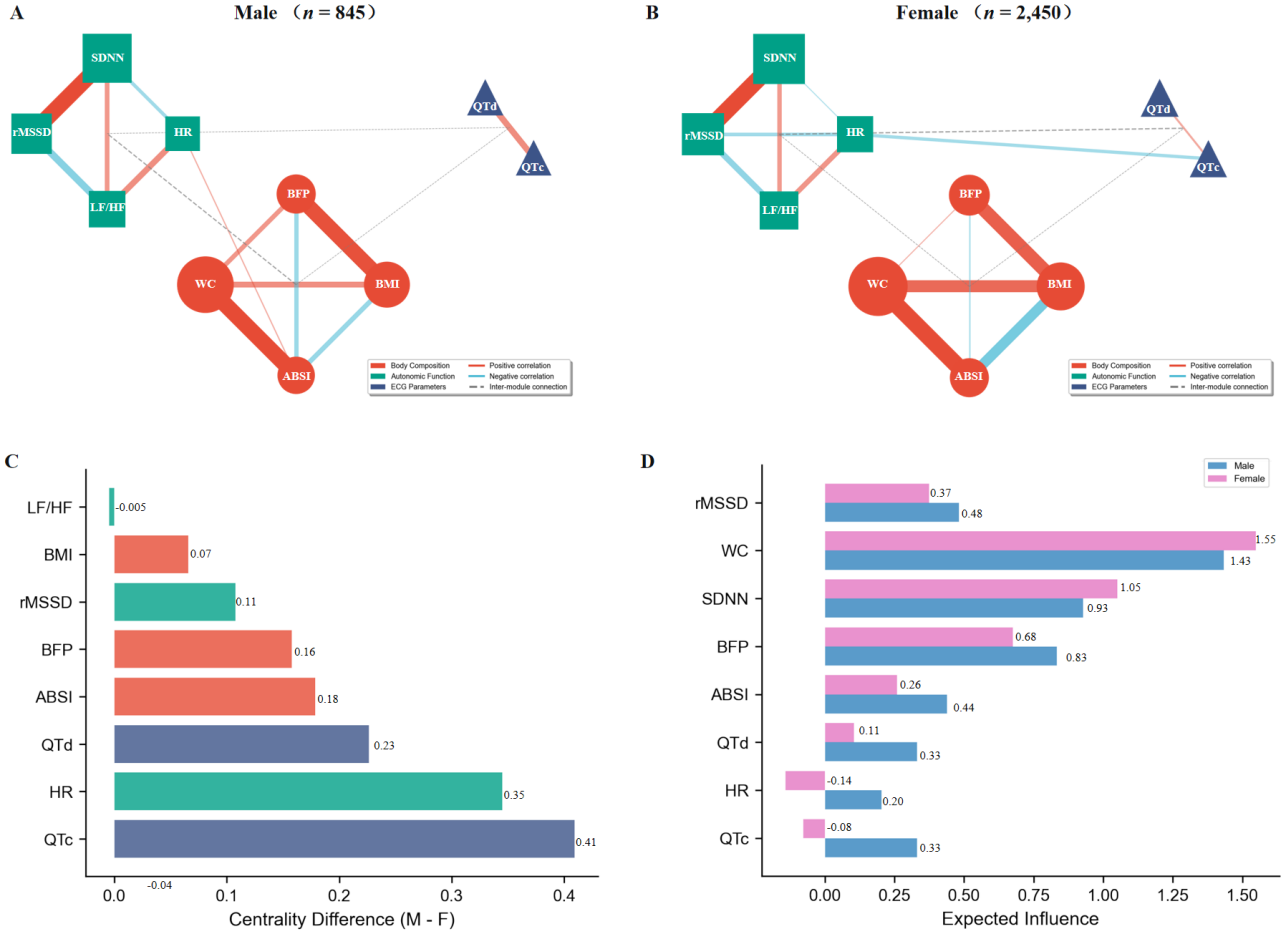
Sex dimorphism in physiological network organization. **(A)** Male-specific network topology with enhanced ECG module connectivity. Only edges with absolute partial correlation > 0.01 are displayed. Edge visualization follows the same conventions as **Figure 1A**. **(B)** Female-specific network exhibiting distinct autonomic-ECG module integration. Network visualization conventions identical to **Figure 1A**. **(C)** Centrality differences (Male - Female) for key nodes, with HR and QTc showing higher centrality in males. **(D)** Butterfly plot comparing node expected influence between sexes, revealing sex-specific hub patterns. WC, Waist Circumference; BMI, Body Mass Index; BFP, Body Fat Percentage; ABSI, A Body Shape Index; ECG, Electrocardiographic; HR, Heart Rate; SDNN, Standard Deviation of NN intervals; rMSSD, Root Mean Square of Successive Differences; LF, Low Frequency power; HF, High Frequency power; QTd, QT dispersion; QTc, Corrected QT interval.

Sex dimorphism was tested by constructing sex-specific networks using a balanced bootstrap resampling method (100 iterations; **Figures 2A, B**; **Supplementary Table 3**). The Network Comparison Test indicated that although the global network structure was highly similar between sexes (similarity coefficient = 0.931) and there was no significant difference in global strength (median difference = 0.039, empirical *p* = 0.730; **Table 2**), significant sex dimorphism was observed at the module and system integration levels. Module strength analysis (**Table 2**) showed that the internal connection strength of the ECG module in males (0.331) was 3.123 times greater than that in females (0.106; empirical *p* < 0.001). Although the strengths of the Body Composition and Autonomic Function modules were numerically similar between sexes, their differences were also statistically significant (empirical *p* < 0.001). Analysis of between-module connectivity further revealed differences in system integration pathways (**Table 2**). A significant ‘Autonomic Function–ECG Parameters’ between-module connection was present in females (connection strength = 0.023), whereas this connection was nearly absent in males (0.000). Conversely, the ‘Body Composition–Autonomic Function’ connection was stronger in males (Male: 0.004; Female: 0.0005). Direct ‘Body Composition–ECG Parameters’ connections were close to zero in both groups. Regarding node centrality, after FDR correction, no nodes showed statistically significant sex differences in Expected Influence (all *q* > 0.05). However, HR and QTc showed trends toward higher centrality in males (differences of 0.345 and 0.409, respectively). A centrality butterfly plot (**Figure 2D**) visually illustrates these sex-specific patterns.

**Table 2:** Sex differences in physiological network topology and centrality.

Metric		Male	Female	Difference	Empirical p	q
Global Topology	Global Strength	6.211	6.172	0.039	0.73	–
	Global Efficiency	0.832	0.785	0.046	0.93	–
	Network Similarity	–	–	0.931	–	–
Module Strength	Body Composition	0.458	0.479	-0.021	< 0.001	–
	Autonomic Function	0.335	0.323	0.012	< 0.001	–
	ECG Parameters	0.331	0.106	0.226	< 0.001	–
Key Node Centrality (EI)	HR	0.203	-0.142	0.345	0.48	0.6
	QTc	0.331	-0.078	0.409	0.41	0.6
	SDNN	0.928	1.052	-0.124	0.49	0.6
	WC	1.433	1.548	-0.115	0.54	0.6

Global strength difference calculated as Male - Female from 100 bootstrap resamples. Module strength ratios calculated as Male/Female. Empirical p-values derived from permutation tests (1000 permutations). Centrality differences represent mean differences in expected influence (Male - Female) across bootstrap resamples. Negative expected influence values indicate that the node’s connectivity profile is less central compared to the network average, not an absence of importance.q-values represent FDR-adjusted p-values using Benjamini-Hochberg procedure (correcting for 10 nodes). EI, Expected Influence; WC, Waist Circumference; ECG, Electrocardiographic; HR, Heart Rate; SDNN, Standard Deviation of NN intervals; QTc, Corrected QT interval; False Discovery Rate; CI, Confidence Intervals.

### 3.4 Obesity effects analysis

**Figure 3:**
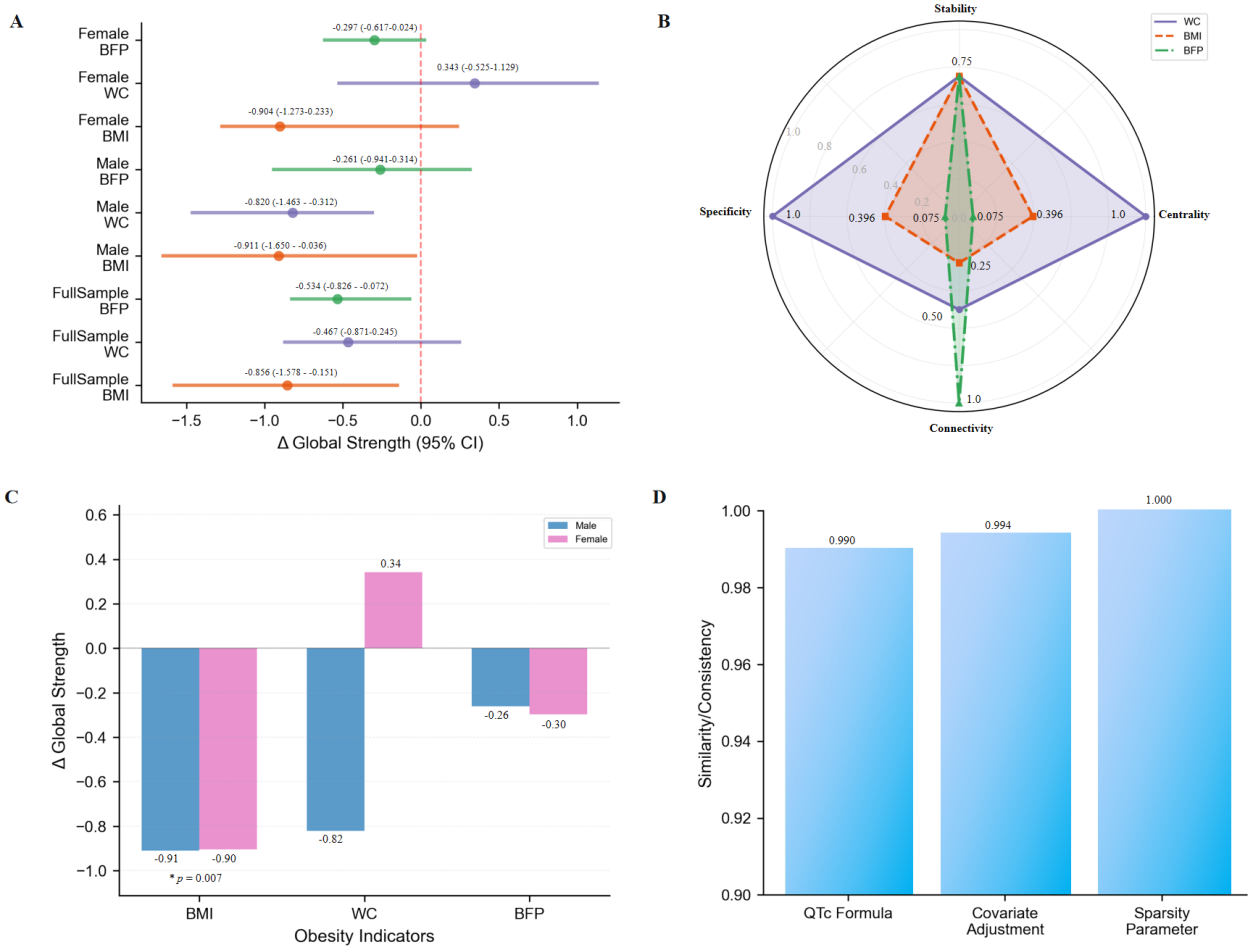
Obesity effects and sex-specific network alterations. **(A)** Forest plot of obesity effects on global network strength across three obesity indicators (BMI, WC, BFP) and sex strata. Negative values indicate network strength reduction in obesity. Error bars represent 95% CI from bootstrap analysis.For detailed sample sizes of participants with obesity in each subgroup, see **Table 3**. **(B)** Radar chart comparing multi-dimensional characteristics of obesity indicators. Dimensions: Centrality (expected influence, reflecting hub status), Stability (bootstrap stability coefficient, CSC = 0.750), Specificity (uniqueness relative to other obesity indicators), Connectivity (number of significant network connections). ABSI is included for network topology comparison but excluded from obesity grouping analysis due to lack of established thresholds. **(C)** Sex × obesity interaction effects on global network strength, suggesting potential gender-specific patterns (uncorrected *p* = 0.007 for BMI interaction, FDR-corrected *q* = 0.065). **(D)** Methodological sensitivity analysis confirming robustness of findings to QTc formula, covariate adjustment, and sparsity parameter choices. WC, Waist Circumference; BMI, Body Mass Index; BFP, Body Fat Percentage; ABSI, A Body Shape Index; QTc, Corrected QT interval; CSC, Correlation Stability Coefficient; FDR, False Discovery Rate; CI, Confidence Intervals.

The impact of obesity status on the multi-system physiological network exhibited indicator-specific and sex-dimorphic patterns (**Figure 3A**; **Table 3**). The forest plot (**Figure 3A**) showed that obesity defined by BMI led to a significant reduction in global network strength (Δ strength = -0.856, 95% CI: -1.578 to -0.151). Obesity defined by BFP also resulted in a significant decrease in network strength (Δ strength = -0.534, 95% CI: -0.826 to -0.072). In contrast, the effect of WC-defined obesity was not statistically significant (Δ strength = -0.467, 95% CI: -0.871 to 0.245). Sex-stratified analysis (**Table 3**) further revealed distinct sex-specific patterns: individuals with obesity according to all three indicators in males consistently exhibited reduced network strength, whereas those with WC-defined obesity in females showed a positive difference in network strength (Δ = +0.343), although the 95% confidence interval included zero (95% CI: -0.525 to 1.129), indicating statistical non-significance. A multi-dimensional comparison radar chart of the obesity indicators (**Figure 3B**) provided a mechanistic explanation for this heterogeneous effect, showing that WC performed optimally in centrality and specificity dimensions. Interaction analysis (**Figure 3C**; **Table 4**) indicated that the sex × BMI-obesity interaction was significant before multiple testing correction (*β* = -0.416, *p* = 0.007) but became non-significant after FDR correction (*q* = 0.065). Interactions involving age and TVED with obesity status were not significant (all *q* > 0.05).

**Table 3:** Obesity effects on physiological network topology metrics.

Analysis level	Obesity indicator	Obesity(n)	Normal (n)	ΔGlobal strength(95% CI)	ΔGlobal efficiency (95% CI)
Full Sample	Obesity BMI	392	2, 903	-0.856 (0.151 to 1.578)	-0.230 (0.052 to 0.292)
Full Sample	Obesity WC	413	2, 882	-0.467 (-0.245 to 0.871)	-0.104 (0.017 to 0.226)
Full Sample	Obesity BFP	934	2, 361	-0.534 (0.072 to 0.826)	-0.115 (0.013 to 0.226)
Male	Obesity BMI	150	695	-0.911 (0.036 to 1.650)	-0.169 (-0.104 to 0.405)
Male	Obesity WC	213	632	-0.820 (0.312 to 1.463)	-0.181 (-0.182 to 0.398)
Male	Obesity BFP	248	597	-0.261 (-0.314 to 0.941)	+0.181 (-0.301 to 0.320)
Female	Obesity BMI	242	2, 208	-0.904 (-0.233 to 1.273)	-0.378 (-0.057 to 0.415)
Female	Obesity WC	200	2, 250	+0.343 (-1.129 to 0.525)	-0.322 (-0.159 to 0.378)
Female	Obesity BFP	686	1, 764	-0.297 (-0.024 to 0.617)	-0.271 (-0.134 to 0.364)

ΔGlobal Strength = Obesity group - Normal weight group. Negative values indicate network strength reduction in obesity. 95% CI calculated from 1000 bootstrap resamples. Obesity definitions: BMI ≥28 kg/m² (both sexes); WC: male ≥90cm, female ≥85cm; BFP: male ≥25%, female ≥30%. Sample sizes reflect complete cases after quality control. WC, Waist Circumference; BFP, Body Fat Percentage; BMI, Body Mass Index; CI, Confidence Intervals.

**Table 4:** Interaction effects between obesity and covariates.

Obesity indicator	Sex interaction			Age interaction			TVED interaction		
	β	p	q	β	p	q	β	p	q
Obesity BMI	-0.416	0.007	0.065	0.107	0.14	0.315	-0.001	0.677	0.762
Obesity WC	0.138	0.364	0.546	0.114	0.109	0.315	0	0.875	0.875
Obesity BFP	0.245	0.055	0.248	0.067	0.24	0.431	-0.001	0.663	0.762

Interaction effects tested using linear models with global network strength as dependent variable. Sex coded as 1 = male, 0 = female; obesity coded as 1=obesity, 0=normal. *β* coefficients represent the interaction term effect size. p-values from two-sided t-tests, q-values represent FDR-adjusted p-values using Benjamini-Hochberg procedure (correcting for 9 tests: 3 obesity indicators × 3 covariates). Statistical significance: *p* < 0.05, *q* < 0.05. WC, Waist Circumference; BFP, Body Fat Percentage; BMI, Body Mass Index; TVED, Total Valid Exercise Distance; FDR, False Discovery Rate.

### 3.5 Sensitivity analysis

Sensitivity analyses (**Figure 3D**; **Table 5**) confirmed the robustness of the main findings. Comparison of QTc formulas showed that networks derived using the Framingham and Bazett formulas were highly similar (network similarity = 0.990), with minimal difference in global strength (absolute difference = 0.020, relative difference = 0.32%). Sensitivity analysis of covariate adjustment strategies indicated that global network strength remained stable across different covariate sets (range: 6.173–6.212), with the number of edges constant (26 edges). Sensitivity analysis of the network sparsity parameter showed that varying the EBICglasso regularization parameter had limited impact on network topology. In simplified networks containing only a single obesity indicator, WC maintained positive connectivity with physiological subsystems (Expected Influence = 0.099), outperforming BMI (0.059) and BFP (−0.138; **Supplementary Table 4**). The observed modularity (Q = 0.130) was higher than the mean of degree-preserving random networks (0.105, *p* = 0.191), and the three-community structure remained stable under bootstrap resampling (CSC = 0.75; **Supplementary Table 5**). A summary of key findings across all analyses is presented in **Table 6**.

**Table 5:** Sensitivity analysis of key methodological choices.

Analysis type	Comparison	Metric	Value	Interpretation
QTc Formula	Framingham vs Bazett	Global Strength Difference	0.02	Negligible
		Network Similarity	0.99	Highly similar
		Relative Difference (%)	0.32%	Minimal impact
Covariate Strategy	No adjustment	Global Strength	6.212	Robust
	Basic adjustment	Global Strength	6.207	Robust
	Full adjustment	Global Strength	6.173	Robust
Sparsity Parameter	EBIC γ = 0	Global Strength	6.212	Insensitive
	EBIC γ = 0.5	Global Strength	6.212	Insensitive
		Number of Edges	26	Consistent
Variable Set Composition	WC-only simplified network	WC Expected Influence	0.099	—
	BMI-only simplified network	BMI Expected Influence	0.059	—
	BFP-only simplified network	BFP Expected Influence	−0.138	—
Modularity Null-Model	Observed vs 1, 000 random networks	Observed Q	0.130	—
		Random Q, mean	0.105	—
		Empirical p-value	0.191	—

QTc formula comparison based on 3295 participants with both Framingham and Bazett calculations. Network similarity calculated as Pearson correlation between network edge weights. Covariate strategies: no adjustment (none), basic (Time period + Age), full (Time period + Age + TVED). Sparsity parameter *γ* = 0 indicates no additional sparsity constraint beyond EBICglasso default. Variable set sensitivity compares simplified 7-node networks each containing a single obesity indicator plus six autonomic and cardiac variables. Modularity null-model uses degree-preserving edge rewiring with Louvain community detection. QTc, Corrected QT interval; TVED, Total Valid Exercise Distance; EBIC, Extended Bayesian Information Criterion.

**Table 6:** Summary of key findings.

Domain	Main finding	Effect size	Statistical significance
Network Structure	Three descriptive functional communities	CSC = 0.75	p = 0.191 vs null model; bootstrap stable
Gender Differences	Overall network similarity high	Similarity = 0.931	p = 0.730 (strength)
	ECG module stronger in males	Strength ratio = 3.123	p < 0.001
Obesity Effects	Global strength reduction	Δ = -0.856 (BMI)	95% CI: 0.151-1.578
	Sex-specific patterns	Male: strength ↓ Female: efficiency ↓	Sex interaction p = 0.007
Methodological Robustness	QTc formula insensitivity	Difference = 0.32%	Similarity = 0.990
	Covariate strategy robustness	Range = 6.173-6.212	Edges constant at 26

Effect sizes and statistical significance summarized from primary analyses. Modularity Q value indicates quality of community detection (range: 0-1). Strength ratios for gender differences in module connectivity. ΔGlobal strength values represent obesity vs normal group differences. Sensitivity differences expressed as absolute values. p-values for interaction effects are uncorrected; FDR-corrected q-values are reported in **Table 4**. All findings robust to methodological variations as shown in **Table 5**. ECG, Electrocardiographic; QTc, Corrected QT interval; BMI, Body Mass Index; FDR, False Discovery Rate.

## 4 Discussion

Our application of Network Physiology in healthy Chinese youth delineates a stable, modular architecture integrating body composition, autonomic function, and cardiac electrophysiology. This map reveals that WC serves as the most central node within this network, reflecting its strong conditional associations with both body composition and physiological subsystems. More importantly, the network’s topology is not uniform but is distinctly shaped by both sex and the specific adiposity indicator examined. Although global network strength was comparable between sexes, we observed divergent patterns of system integration: males exhibited stronger within-system connectivity, particularly among ECG parameters, whereas females demonstrated more robust cross-system coupling between autonomic and cardiac electrophysiological functions. Concurrently, obesity exerted indicator-specific effects, with BMI and BFP generally attenuating network connectivity, while WC-defined obesity showed a unique, non-attenuating pattern, especially in females. These primary findings, which address our aim to characterize the system’s inherent organization and its modifiers, were robust across sensitivity analyses.

### 4.1 Modularity and highly central nodes in youth physiological networks

The observed three-module structure demonstrates that body composition, autonomic, and cardiac electrophysiological functions operate as distinct yet physiologically coupled subsystems in youth. This modularity aligns with principles of specialized physiological organization reported in other systems (Barajas-Martínez et al., 2020). A key finding was the heterogeneous influence of different adiposity measures within this architecture. WC emerged as the most central node, with significantly higher expected influence than BMI, BFP, or ABSI. This centrality is consistent with WC’s role as a surrogate for visceral adiposity, which is associated with multiple physiological systems through mechanisms such as meta-inflammation and autonomic dysregulation (Christakoudi et al., 2020). The strength of the WC-ABSI connection within the body composition module further highlights the distinct information captured by central adiposity indices. In contrast, BFP was the sole obesity indicator directly linked to QTc, suggesting that systemic fat accumulation may be associated with cardiac repolarization through distinct mechanisms, such as lipotoxicity (Omran et al., 2018), potentially independent of the autonomic pathways. The relatively limited role of ABSI in our network suggests that in young, relatively healthy cohorts, its association with metabolic dysregulation may not yet be fully manifest (Ji et al., 2018), or that its predictive power for mortality operates through pathways not captured by our chosen physiological subsystems.

### 4.2 Sex-specific patterns of physiological system integration

Our analysis revealed distinct sex differences in how physiological systems covary, despite similar global network structures. Males displayed stronger intrinsic connectivity within the ECG module, whereas females exhibited stronger coupling between the autonomic and ECG modules. This topological divergence may reflect established sex differences in autonomic nervous system baseline regulation. Males typically have higher sympathetic tone, which may be associated with stronger within-ECG module connectivity and concurrently weaker autonomic-cardiac conditional associations (weaker autonomic-ECG coupling) (Porta et al., 2017; Hamidovic et al., 2020). This is consistent with a physiology where sympathetic dominance promotes uniform ventricular repolarization patterns. Conversely, the higher vagal tone prevalent in females (Koenig and Thayer, 2016) may be associated with stronger cross-module connectivity between the autonomic and cardiac systems, resulting in the observed stronger cross-module integration. This female-predominant pathway may facilitate rapid homeostatic adjustment via vagal withdrawal. Thus, the distinct network topologies may reflect sex-specific patterns of physiological organization at rest, with potential implications for how each sex responds to physiological stress.

### 4.3 Adiposity indicators are differentially associated with network topology

The association between obesity and network topology was not uniform but demonstrated clear indicator and sex specificity. The general attenuation of network strength associated with BMI and BFP is consistent with broad physiological differences associated with obesity (Ivanov et al., 2016; Blüher, 2019). In contrast, WC-defined obesity did not significantly reduce global strength and even showed a trend towards preserved connectivity in females. This suggests that the physiological correlates of central adiposity, particularly in its early stages, may differ from that of overall obesity. The unique BFP-QTc connection further supports the concept of indicator-specific pathways to electrical risk (Omran et al., 2018). Collectively, these findings support the integration of multi-dimensional adiposity assessment in clinical practice (Aguas-Ayesa et al., 2024; Sweatt et al., 2024), and align with recent calls for a new framework in obesity diagnosis that moves beyond BMI-centric approaches (Busetto et al., 2024). The trend of preserved network strength with high WC in females, while preliminary due to sample size constraints, suggests potential sex-specific mechanisms (de Koning et al., 2007). Estrogen-mediated fat distribution (subcutaneous vs. visceral) (Morselli et al., 2016; Loh et al., 2022; Steiner and Berry, 2022) and greater autonomic reserve capacity in females (Wiley et al., 2020) might be associated with the observed sex-specific network patterns (Stevens et al., 2010). This hypothesis, which posits that the female physiological network may show a distinct pattern of association with central obesity, requires explicit testing in future longitudinal studies that track the transition from health to disease.

### 4.4 Perspectives on system-level physiological organization

The high centrality of WC suggests its physiological relevance extends beyond that of BMI, as it shows the strongest conditional associations with multiple physiological subsystems (Bashan et al., 2012). The sex-dimorphic network patterns suggest hypothesis-driven studies of risk assessment: in males, ECG parameters show stronger within-module connectivity, whereas in females, autonomic-cardiac coupling deserves attention (Liu et al., 2024). Our findings suggest the potential utility of a multi-system assessment paradigm (Elmaleh-Sachs et al., 2023) complementing single-threshold screening. Future integration of such network metrics with data from wearable devices could facilitate dynamic monitoring for early cardiovascular risk stratification, advancing towards the vision of a personalized “physiolome” (Ivanov, 2021).

### 4.5 Limitations and future directions

Several limitations merit consideration. (1) The cross-sectional design precludes causal inference regarding the directionality between obesity and network topology. The observed network represents a map of conditional associations that generates hypotheses for future mechanistic studies, not evidence of causal or regulatory pathways. Longitudinal studies tracking network reorganization during weight change are required to determine whether the observed topological alterations precede or follow adiposity accumulation. (2) The absence of inflammatory (e.g., CRP, IL-6) and detailed metabolic biomarkers limits our ability to elucidate the molecular mediators underlying the observed network connections (Jarczok et al., 2014; Williams et al., 2019). For instance, the direct partial correlation between BFP and QTc may reflect lipotoxic effects on ventricular repolarization, but without concurrent inflammatory or metabolic data, this mechanistic interpretation remains speculative. (3) The compact size of our network (10 nodes) imposes inherent topological constraints on modularity estimation. While the three-community structure demonstrated high bootstrap stability (CSC = 0.75), the absolute modularity (Q = 0.130) did not significantly exceed random expectation (*p* = 0.191), a finding attributable to the limited dynamic range of modularity in small networks rather than an absence of functional organization. (4) The imbalanced sex ratio (845 males vs. 2, 450 females) reflects the demographics of the source institution, where female students constitute the majority of health science programs. No *a priori* sample size calculation was performed; the sample was determined by the available population within a fixed academic term, and *post-hoc* power analysis confirmed adequate statistical power. Although balanced bootstrap resampling was employed in the Network Comparison Test to mitigate this imbalance, sex-stratified analyses involving smaller subgroups should be interpreted with appropriate caution. (5) While some observed effect sizes were modest (e.g., between-module connectivity strengths), their cumulative impact on global network topology may have physiological relevance. The clinical significance of small effects in densely connected physiological networks warrants further investigation. (6) Our cohort of Chinese university students may limit generalizability to other age groups, ethnicities, or populations with established disease. Independent replication in diverse populations and prospective longitudinal cohorts is necessary to validate and extend these findings.

## 5 Conclusion

In conclusion, this study maps the multi-system physiological network in healthy Chinese youth, revealing a structure that differs markedly by both sex and the specific dimension of adiposity considered, thereby providing a system-level perspective on early risk. We identify WC as the most central node, underscoring the particular relevance of central adiposity in system-wide physiological coordination. We demonstrate that males and females exhibit distinct patterns of system integration, likely reflecting sex-specific autonomic regulation, with males showing tighter within-system and females stronger between-system coordination. Finally, we show that the association between obesity and network topology is not uniform: overall adiposity (BMI, BFP) is associated with weaker network connectivity, whereas central adiposity (WC) exhibits a more complex, sex-dependent pattern. These findings provide a system-level framework for studying cardiometabolic risk from early life and highlight the potential of integrative physiological network analysis to reveal how multiple systems covary in health, complementing traditional approaches that focus on isolated risk factors.

## Data Availability

The original contributions presented in the study are included in the article/**Supplementary Material**. Further inquiries can be directed to the corresponding author.
