# Supplementary figures and images for: Central adiposity and sex are associated with the integrative architecture of physiological networks in healthy youth: a cross-sectional study in a cohort of Chinese college students
Source: Front Physiol. 2026 Jul 16;17:1859147. doi: 10.3389/fphys.2026.1859147 (PMC13422149; doi:10.3389/fphys.2026.1859147)

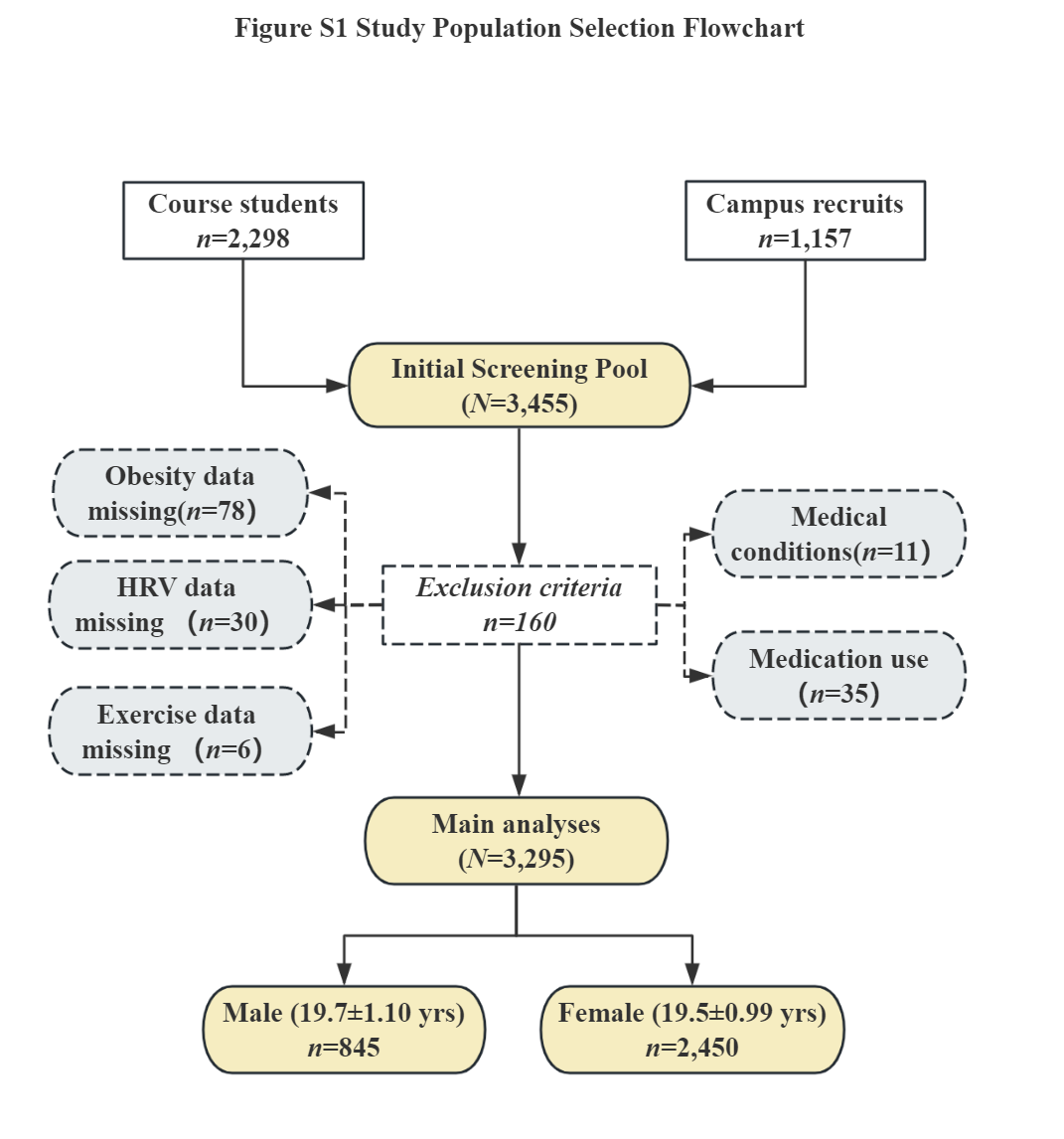

Supplement: Supplementary file 1 [file Image1.tif]
